# Insights into the fine-scale habitat use of Eurasian Water Shrew (*Neomys fodiens*) using radio tracking and LiDAR

**DOI:** 10.1093/jmammal/gyae146

**Published:** 2025-01-10

**Authors:** Towi A W van der Putten, Joris J F Verhees, Zsofia Koma, Paul H van Hoof, Dirk Heijkers, Willem F de Boer, Helen J Esser, Gert Hoogerwerf, Pim Lemmers

**Affiliations:** Natuurbalans–Limes Divergens, Toernooiveld 1, 6525 ED Nijmegen, The Netherlands; Wildlife Ecology and Conservation Group, Department of Environmental Sciences, Wageningen University, Droevendaalsesteeg 3a, 6708 PB Wageningen, The Netherlands; Natuurbalans–Limes Divergens, Toernooiveld 1, 6525 ED Nijmegen, The Netherlands; Department of Biology, Center for Sustainable Landscapes Under Global Change, Aarhus University, Aarhus 8000, Denmark; Norwegian Institute of Bioeconomy Research (NIBIO), Høgskoleveien 8, 1433 Ås, Norway; Natuurbalans–Limes Divergens, Toernooiveld 1, 6525 ED Nijmegen, The Netherlands; Natuurbalans–Limes Divergens, Toernooiveld 1, 6525 ED Nijmegen, The Netherlands; Wildlife Ecology and Conservation Group, Department of Environmental Sciences, Wageningen University, Droevendaalsesteeg 3a, 6708 PB Wageningen, The Netherlands; Wildlife Ecology and Conservation Group, Department of Environmental Sciences, Wageningen University, Droevendaalsesteeg 3a, 6708 PB Wageningen, The Netherlands; Natuurbalans–Limes Divergens, Toernooiveld 1, 6525 ED Nijmegen, The Netherlands; Natuurbalans–Limes Divergens, Toernooiveld 1, 6525 ED Nijmegen, The Netherlands; Radboud University, Radboud Institute for Biological and Environmental Sciences, Department of Animal Ecology and Physiology, P.O. Box 9100, 6500 GL Nijmegen, The Netherlands

**Keywords:** airborne laser scanning (ALS), habitat use, remote sensing, Soricidae, species distribution model (SDM), vegetation structure

## Abstract

The Eurasian Water Shrew (*Neomys fodiens*) is one of the largest shrew (Soricidae) species in Eurasia. In Western Europe, this semiaquatic species often occurs in riparian and marshland habitats that have a high degree of naturalness, but is being threatened by habitat degradation and other anthropogenic factors. The species mostly occurs in low abundance and is elusive. Therefore, understanding its habitat use is challenging, yet imperative for establishing species-specific conservation measures. Technological developments in radio tracking and high-resolution remote sensing such as Light Detection And Ranging (LiDAR) now enable the quantification of ecological niches and provide insight into habitat requirements for a species. Here, we combined radio tracking and LiDAR to quantify habitat use by Eurasian water shrews. Alongside a lowland brook in the Netherlands, 20 individuals were tracked between September and October 2022, resulting in 332 unique locations of Eurasian water shrews. For each of these locations, 11 LiDAR-derived variables were calculated and subsequently analyzed in a species distribution model (SDM). The SDM yielded a model with a high accuracy (predictive performance AUC = 0.93). The variable of highest importance was dense and relatively short vegetation <1 m, which had a positive effect on Eurasian Water Shrew occurrence. Open areas seem to be avoided. Vegetation of heights between 1 and 15 m were found to be less important for the occurrence. The probability of occurrence decreased with increasing distance to water, indicating that the species occurs in the proximity of water, although vegetation-related variables were more important. The obtained detailed knowledge of fine-scale habitat use can be used to improve habitat conservation, restoration, and management for the species. Combining radiotelemetry data with LiDAR data is a promising approach to identifying species–habitat relationships of elusive species such as the Eurasian Water Shrew.

## Teaser

The elusive Eurasian Water Shrew occurs in low abundances. Understanding its habitat use is imperative for conservation. Combining radio tracking and LiDAR demonstrated that dense and short vegetation had a positive effect on its occurrence but open areas are avoided.

Stream valleys in Northwestern Europe host a rich biodiversity driven by the dynamic nature of ecosystems, in which flowing water and natural disturbances such as occasional flooding create a wide variety of habitats and resources (Tornwall et al. 2015; Fraaije et al. 2019; Verdonschot and Verdonschot 2022). During the past centuries, stream valleys have been severely impacted by anthropogenic influences such as channelization, dam construction, and agricultural intensification (Fraaije et al. 2019; Mazor et al. 2022). This history of disturbance led to the degradation, desiccation, fragmentation, and loss of stream valley habitats. Furthermore, climate change-driven droughts have put additional pressure on stream valleys and their associated species (Boulton 2003). One species that is particularly associated with natural stream valleys is the Eurasian Water Shrew (*Neomys fodiens;*  Rychlik 2000; Carter and Churchfield 2006). This solitary species occurs in low densities in well-developed rugged riparian vegetation or marsh forests, always in the proximity of freshwater habitats (Van Bemmel and Voesenek 1984; Rychlik 2000; Champneys 2012; Sheftel 2018). Studying the habitat use of this small and elusive species has proven to be difficult (French et al. 2001; Carter and Churchfield 2006; Champneys 2012). However, in order to carry out appropriate habitat conservation and restoration measures, it is essential to obtain detailed knowledge about its fine-scale habitat use (Champneys 2012).

Previous studies on habitat use of Eurasian Water Shrew were conducted under controlled conditions (Rychlik 1997), or based on field observations that were constrained by technological possibilities at that time (Stickel 1954; Cantoni 1993; Rychlik 2000; French et al. 2001). Recent technological developments in the fields of radio tracking and remote sensing now enable the study of fine-scale habitat use by small species at unprecedented detail (e.g., Gubert et al. 2023). For example, Light Detection And Ranging (LiDAR) remote sensing measurements—obtained using airborne laser scanning (ALS)—yield high-resolution 3D measurements of landscape structures from which variables such as elevation, water surface, and vegetation densities can be calculated (e.g., Valbuena et al. 2020; De Vries et al. 2021). From these LiDAR measurements, habitat structure parameters (i.e., variables) can be calculated. These variables can then be used to quantify the ecological niche and provide insights into habitat use of the species (Davies and Asner 2014; Simonson et al. 2014; Gubert et al. 2023).

This study aimed to identify the fine-scale habitat and vegetation structures that determine the habitat use of Eurasian water shrews by combining robust telemetry data with fine-scale landscape variables obtained from LiDAR surveys. Combining radio tracking with LiDAR data to quantify habitat use has recently been used for bats, birds, and dormice (Garabedian et al. 2017; Carr et al. 2018; Goodwin et al. 2018; Gubert et al. 2023). Previous studies showed that Eurasian water shrews prefer dense herbal cover (Greenwood et al. 2002; Keckel et al. 2014), and little tree cover (Greenwood et al. 2002). We therefore hypothesized (H1) that a short and dense vegetation has a positive effect on Eurasian Water Shrew habitat use. Because short dense vegetation is often found at the forest edge, we also hypothesized (H2) that a larger forest edge has a positive relationship with Eurasian Water Shrew habitat use. Depending on the area studied, the diet of Eurasian water shrews can consist predominantly of aquatic prey (Buchalczyk and Pucek 1963; Wołk 1976; Niethammer 1978; Dupasquier and Cantoni 1992) or partly so (e.g., Gastropoda, Trichopteran larvae, and Hirudinea; Rychlik 1997, 2000). Due to their semiaquatic lifestyle (Mendes-Soares and Rychlik 2008; Keckel et al. 2014) and their occurrence in the proximity of water (Van Bemmel and Voesenek 1984; Churchfield and Rychlik 2006; Champneys 2012; Sheftel 2018), we hypothesized (H3) that an increased distance to water has a negative effect on the probability of occurrence of Eurasian Water Shrew.

## Materials and methods.

### Study area.

**Fig. 1.**
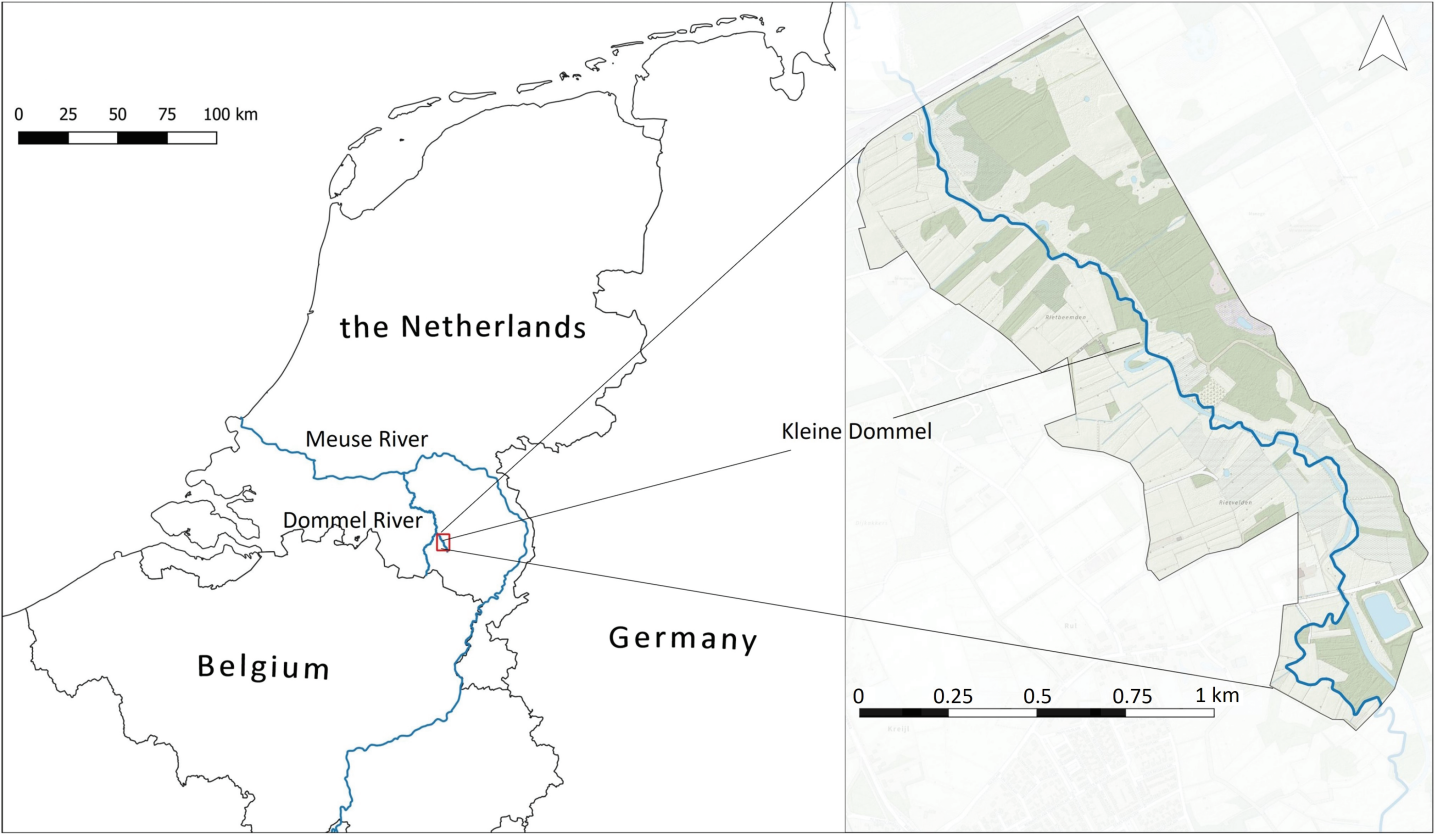
A map of the study area alongside the lowland brook (Kleine Dommel), The Netherlands.

This study was carried out alongside a lowland brook (Kleine Dommel) in the Netherlands near the city of Eindhoven (Fig. 1). The regular water level is approximately 40 to 80 cm. At the time of the study, there was a drought and the water level was estimated at 10 to 40 cm. Between 2017 and 2022, a part of this brook was rehabilitated to a natural meandering flow and the surrounding area was designed as a nature reserve (Winkel-Bootsma 2014). As a result, the study area is rich in habitats consisting of different meadows, riparian vegetation, shrubs, reedbeds, and forests (Supplementary Data SD1). The banks of the brook were natural, with a gentle angle and partly covered with several species of herbaceous vegetation and Common Reed (*Phragmites australis*). A recent study demonstrated that Eurasian water shrews are relatively abundant in the study area (Verhees et al. 2024).

### Live trapping, transmitter attachment, and radio tracking.

Eurasian water shrews were captured using Heslinga live traps, which were placed in a total of 20 transects throughout the study area. Transect locations were selected based on the known occurrence of Eurasian water shrews (NDFF 2023) or suitable habitats based on expert judgment. Live trapping was conducted in 2 periods: from 13 to 22 September 2022 in 10 transects and from 4 to 14 October 2022 in 10 other transects (see Verhees et al. 2024 for the transect details). In each transect, 20 live traps were placed in pairs with 10-m interspacing for a total of 200 live traps per period (Bergers and La Haye 2000; Flowerdew et al. 2004). Live traps were each day refilled with dry hay for nesting material and insulation to minimize mortality risk, and baited with a handful of live mealworms, muesli, and carrots (Keckel et al. 2014). To allow animals to get used to the traps, we used a prebaiting period of 3 days during which the traps were inactive. After activation, the traps were checked twice per day during dawn and dusk (Bergers and La Haye 2000), for a maximum duration of 4 days. After an animal was caught, live traps were replenished with fresh bait and dry hay.

Twenty caught Eurasian water shrews were fitted with a Holohil Systems Ltd BD-2X transmitter (pulse rate 30 ppm). This model was chosen for its light weight of 0.39 g and relatively long lifespan (presumed 21 days). To ensure that a transmitter was less than 5% of body weight, an individual had to weigh at least 7.8 g to be eligible for a transmitter. Hence, each captured Eurasian Water Shrew was weighed and individuals that did not meet this criterion were immediately released at the site of capture. All 20 caught individuals weighed enough to be fitted with a transmitter.

During the transmitter attachment process, Eurasian water shrews were not anesthetized as anesthesia may cause complications for the well-being of an animal (Rychlik et al. 2010). Transmitters were applied *ex situ* by 2 people, one person holding the shrew securely and the second person applying the transmitter. In accordance with Rychlik et al. (2010), transmitters were initially applied by cutting a piece of fur away between the scapula. Then, the transmitter was glued onto the skin with Sauer Hautkleber, after which the remaining fur surrounding the trimmed area was partly glued to the sides of the transmitter to improve the attachment. After 4 animals we noticed that cutting the fur was not necessary, and that moving the fur between the scapula sideways and then gluing the transmitter on the exposed patch of skin resulted in a quicker but just as effective procedure. During the process, the head of the animal was covered to minimize stress. The transmitter attachment process took 15 to 20 min per animal including drying of the glue. To monitor their recovery and correct transmitter attachment, shrews were subsequently placed in a cage, provided with food, water, and nesting material and observed for minimally 3 h before release. All animals were released at the same location as where they were caught within 12 h after capture. ASM guidelines were followed (Sikes et al. 2016). The catching of Eurasian water shrews had been carried out under a Wnb license ODH-2020-00001393, issued by the Network Groene Bureaus. The project was approved by the Radboud University Animal Experiment Committee and was performed under animal experimentation Wod license No. TRC/NVWA/2014/1558 of licensee Natuurbalans–Limes Divergens BV.

Tracking of individuals started the first night after release. This was done with a Followit Y-4FL 4-element Yagi antenna, combined with a Biotrack SIKA Radio tracking receiver. We aimed to obtain as much data as possible from each individual since a previous study revealed that transmitters remained attached for a mean of 2 days (Rychlik et al. 2010). Therefore, we established an intensive monitoring schedule by tracking shrews in teams of 2 people from dusk to dawn (approximately 21:00 to 07:00 h). We later found that the animals were less active after 1:00 to 2:00 h, as also demonstrated by Rychlik (2005). When the distance from an observer to a Eurasian Water Shrew, using triangulation with 3 different angles, was estimated to be 5 m (this distance was found not to disturb the shrews), the first coordinate of the animal’s location was recorded. Then, the animal was continuously tracked and coordinates were recorded every 5 min, yielding a total of 4 positions per individual per 15 min. After 15 min, the next individual was tracked following the same procedure. This procedure continued until all animals were tracked once, after which the first animal was tracked again. This cycle continued as often as possible until dawn. The position of each tracked individual was recorded using a GPS (accuracy 3 m) and saved in the mobile application “WrnPro.” The use of this application allowed for quick and accurate positions during fieldwork and the possibility to (automatically) note additional information such as time of observation, weather circumstances, information on shrew activity (resting, active, foraging), etc. Ultimately, data were downloaded from the application and imported to an Excel file for further data processing.

### LiDAR data processing and analysis.

The Dutch national ALS map AHN3 was used to calculate LiDAR-derived variables (AHN n.d.). AHN is a data set collected and owned by the government (Ministry of Infrastructure and Water Management) and publicly accessible as open data. The LiDAR flight campaign was conducted between 2014 and 2019 under leaf-off conditions in winter (between December and March). LiDAR data were collected in our study area between 30 November and 1 December 2016. A visual comparison of satellite imagery using Google Earth over the past 10 years was conducted to determine if vegetation had changed substantially between the 2016 LiDAR survey and the 2022 fieldwork. The LiDAR data have a mean point density of 6 and 10 pt m^−2^. Ground truthing has been undertaken by AHN to validate the LiDAR data. The overall accuracy of the geolocation is 10 cm horizontally and 5 cm vertically. This accuracy is partly due to GPS coordinates, on which LiDAR data gets mapped. GPS provides the location while LiDAR provides the detailed measurements. The Dutch National ALS data set is classified into ground points, buildings, structures, water, and others, from which vegetation points can be extracted (AHN n.d.). A 5 × 6.3 km section of LiDAR data was downloaded from https://ahn.arcgisonline.nl/ahnviewer from which 3 tiles of 1 × 1 km were extracted that covered the complete study area.

We used the “lidR” package (Roussel et al. 2020) in the R software (R Core Team 2022) for processing the LiDAR data. Tracking of shrews resulted in 1,065 data points (i.e., positions). However, since each position that was recorded within the same 15 min was spatially autocorrelated to some degree, we chose a conservative approach and only used the first out of 4 data points for further analyses (i.e., the first position of a tracked individual). This resulted in a data set of 332 unique data points, each representing a position of an individual Eurasian Water Shrew. Subsequently, within the 3 1 × 1 km tiles that covered the study area, 332 random coordinates were generated and used as background positions for the species distribution modeling. The points were randomly selected using the “sampleRandom” function in the “raster” package (Hijmans 2023).

At each of the total 664 positions, 2 circles with a radius of 10 and 35 m, respectively, were clipped from the LiDAR point cloud to quantify vegetation structure at both the local and landscape level. Subsequently, clips, referred to as “plots,” were spatially normalized using a *k*-nearest neighbor approach with an inverse-distance weighting to account for differences in terrain height (default arguments with *k *= 10 and *P *= 2; Roussel et al. 2020). The normalized plots were clipped again until they had a radius of 5 and 25 m, thus removing the outer normalization buffer. The radius of 5 m was chosen to quantify vertical complexity of the vegetation at the smallest possible level without losing accuracy due to GPS margins. The larger radius of 25 m was chosen to quantify horizontal landscape-level heterogeneity, without the radii of data points overlapping too much. Hence, this resulted in a total of 1,328 normalized individual plots (i.e., 664 plots with a 5-m radius and 664 plots with a 25-m radius) that were used for further calculation of the variables. An additional set of plots was made for the distance to water variable, with 250 and 400 m radii so the distance could be calculated for every data point, even when the distance to water was larger than 5 or 25 m.

In total, we calculated 7 variables that captured local-level vertical complexity of vegetation (i.e., <1 m density, 1 to 5 m density, 5 to 10 m density, >10 m density, vegetation height, structural complexity, and canopy cover) and 4 variables that captured the horizontal landscape-level heterogeneity (i.e., distance to water, open areas, forest edge length, and variation in vegetation height; Table 1). Density is defined as the percentage of the total LiDAR points in a plot, structural complexity is defined as the standard deviation of the height, and variation in vegetation height is defined as the variance in vegetation height within a plot. All variables were selected based on previous Eurasian Water Shrew studies (Wołk 1976; Rychlik 1997; Rychlik 2000; Churchfield and Rychlik 2006; Mendes-Soares and Rychlik 2008; Alejūnas and Stirkė 2010; Panzacchi et al. 2010; Keckel et al. 2014; Stein et al. 2014; Biffi et al. 2019; Table 1).

**Table 1.** LiDAR-derived variables representing different aspects of landscape-level horizontal heterogeneity and local-level vertical complexity. “Variable” lists the LiDAR-derived variables that are calculated for this study, “Method of calculation” describes how the variable was calculated in the statistical analysis program R, “Vegetation layer” refers to which vegetation layer the variable is targeting, “Hypothesis” describes which hypothesis this variable is trying to answer, “Ecological relevance” gives ecological context to why these variables were chosen. The bottom 3 variables were calculated but excluded from the analysis.

Variable [unit]	Description	Method of calculation	Vegetation layer	Hypothesis	Ecological relevance
Landscape-level horizontal heterogeneity					
Open areas [m^2^]	Area in which vegetation does not exceed 0.5 m in height within a 25-m radius.	After rasterization into 1-m^2^ plots, it was determined whether vegetation in the plots exceeded 0.5 m in height. If not, that was counted as open area. All these squares were then summed.	Extent of open vegetation	H1	European water shrews avoided areas with denser herbal coverage (Keckel et al. 2014).
Variation in vegetation height [m]	Variance in vegetation height within a 25-m radius.	After rasterization, a quantile 90% function was carried out on each of the 1-m^2^ plots and the variance was calculated.	All	H1	Heterogeneity in vegetation promotes biodiversity of flora and fauna (Stein et al. 2014). This may also be the case with Eurasian water shrews.
Forest edge length [m]	Length of the total forest edge within a 25-m radius.	After rasterization, forest was defined as vegetation above 3 m. Edges of the 1-m^2^ plots were summed up for each forest plot.	Tree	H2	Eurasian waters shrews were caught in mixed forests (Alejūnas and Stirkė 2010).
Distance to water [m]	Distance to closest water from central coordinates of every data point.	After rasterization into 1-m^2^ plots. Water was characterized by having no LiDAR data. Then the distance to the closest “no data” from the middle of the plot was calculated.	Ground	H3	The diet of Eurasian water shrews consists partly of terrestrial and partly of aquatic prey (Wołk 1976; Rychlik 1997; Churchfield and Rychlik 2006). And Eurasian Water Shrew has well-developed swimming and diving abilities (Mendes-Soares and Rychlik 2008).
Local-level vertical complexity					
Vegetation height [m]	Maximum tree canopy height in a 5-m radius.	Quantile 90% function.	Tall vegetation	H1	Vegetation height can indicate preference of open areas or forests. Old tree stumps were often used by Eurasian water shrews (Rychlik 2000; Biffi et al. 2019).
<1 m density [%]	Density of vegetation between 0 and 1 m from the ground within a 5-m radius.	(LiDAR points below 1 m/Total LiDAR points) × 100.	Herb or grass	H1	Juvenile and subadult Eurasian water shrews prefer a dense herbal cover (Keckel et al. 2014).
1 to 5 m density [%]	Density of vegetation between 1 and 5 m within a 5-m radius.	(LiDAR points between 1 and 5 m/Total LiDAR points) × 100.	Shrub	H1	
5-10 m density [%]	Density of vegetation between 5 and 10 m within a 5-m radius.	(LiDAR points between 5 and 10 m/Total LiDAR points) × 100.	Tree	H1	
Local-level variables not used in the final model					
Structural complexity [m]	Standard deviation of the height.	Standard deviation of the height.	All	H1	Small mammals prefer high structural heterogeneity (Panzacchi et al. 2010).
Canopy cover [%]	Density of the canopy, which is the percentage of vegetation above 3 m.	(LiDAR points above 3 m/Total LiDAR points) × 100.	Tree	H1	A diverse tree canopy results in favorable conditions for small mammals (Panzacchi et al. 2010).
>10 m density [%]	Density of vegetation above 10 m within a 5-m radius.	(LiDAR points above 10 m/Total LiDAR points) × 100.	Tree	H1	Juvenile and subadult Eurasian water shrews prefer a dense herbal cover (Keckel et al. 2014).

### Species distribution modeling.

To analyze the effect and relative importance of the variables (Table 1) for explaining the habitat use of Eurasian water shrews, we used a species distribution model (SDM). The R packages: “lidR,” “rlas” (Roussel and De Boissieu 2022), “raster,” and “future” (Bengtsson 2021) were used to tile and clip LiDAR data from a bigger data set. For the extracting, normalizing, and clipping of LiDAR data plots, the R packages: “lidR” and “raster” were used. Calculating the LiDAR variables was done using the R packages: “lidR,” “rlas,” “tidyverse” (Wickham et al. 2022), “raster,” “rgeos” (Bivand and Rundel 2022), “sdm” (Naimi and Araujo 2016), “usdm” (Naimi et al. 2014), “gridExtra” (Auguie 2017), “dismo” (Hijmans et al. 2022), “Hmisc” (Harrell 2021), “corrplot” (Wei and Simko 2021), “terra” (Hijmans 2022), and “landscapemetrics” (Hesselbarth et al. 2019). The SDMs were made using the R packages: “lidR,” “Hmisc,” “raster,” “tidyverse,” “sdm,” “rgeos,” “dismo,” and “corrplot.” Lastly, the figures were made using the R packages “ggplot2” (Wickham 2016), “gridExtra,” and “sdm.” First, we tested for multicollinearity between variables using a Spearman rank correlation. Variables that were highly correlated (*r* > 0.7) were stepwise removed from the SDM, starting at variables that showed the strongest correlation and least ecological relevance with regard to our field observations (De Vries et al. 2021).

In the SDM, we used the “maximum entropy” algorithm (i.e., Maxent) because of its ability to analyze presence-only data (Phillips et al. 2006, 2017; Elith et al. 2010). The model accuracy was evaluated using area under curve (AUC) values and visual examination of receiver operating characteristics (ROC) curve. AUC values between 0.7 and 0.8 were interpreted as fair, between 0.8 and 0.9 as good, and >0.9 as excellent (Swets 1988). The model was built with default settings and 100 random bootstraps using a subset of 70% of the data, and the remaining 30% to validate the predictive performance during each repetition. For each run, the mean accuracy values and associated standard deviations were reported. The final model was obtained through a backward selection procedure in which the least explanatory variables were stepwise omitted.

To test each hypothesis (Table 1), we extracted the relative variable importance and associated response curves of the variables to predict the probability of occurrence of Eurasian water shrews in relation to these variables. The relative variable importance was assessed using the AUC (Naimi and Araujo 2016). Response curves represent the probability of occurrence in relation to each vegetation variable gradient, while keeping the other variables at their mean (Elith et al. 2005).

## Results

**Fig. 2.**
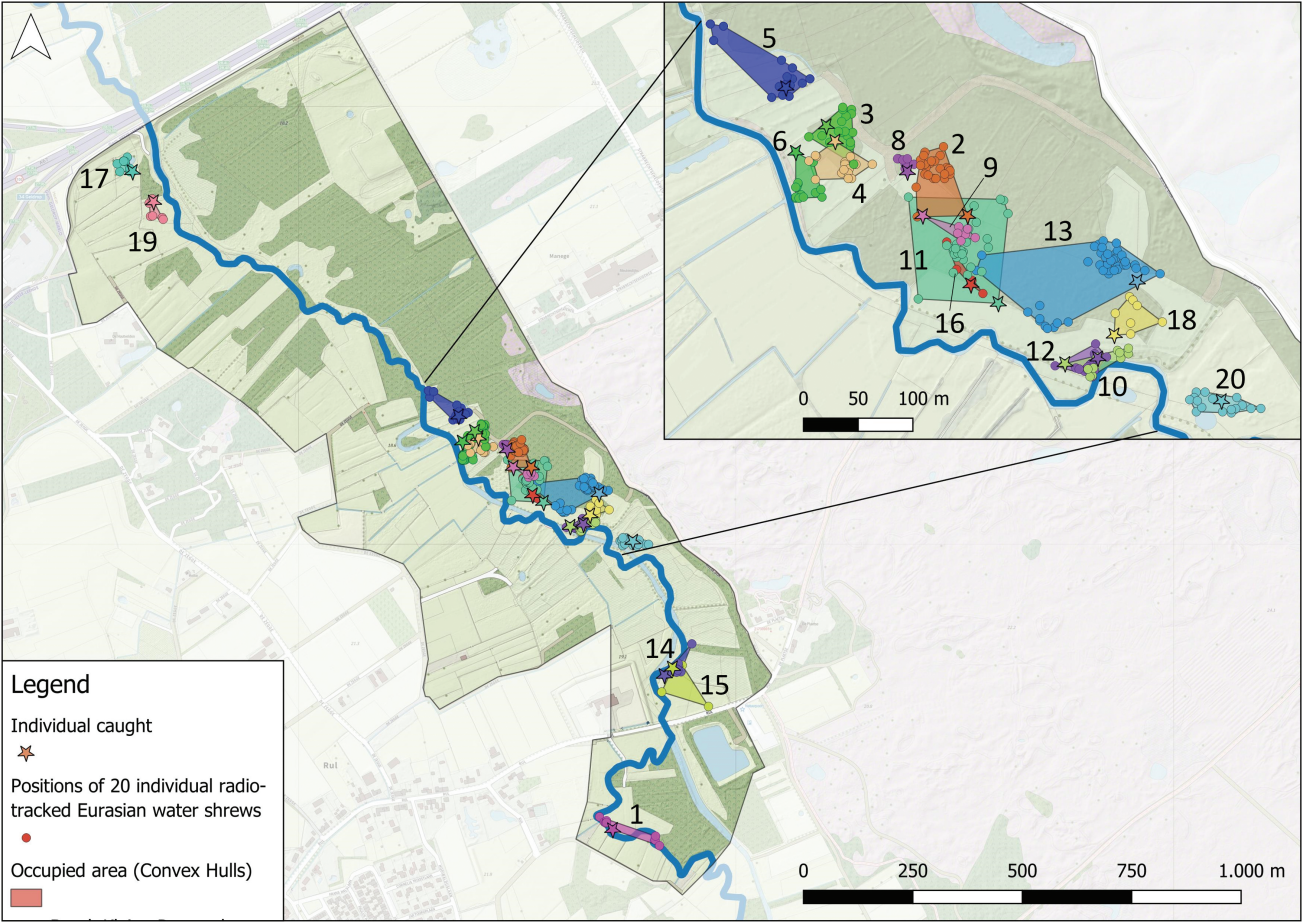
Area used by each Eurasian Water Shrew (*Neomys fodiens*) during the field study. Each color indicates a unique individual (*n =* 20). Individual 7 is not shown because it lacked enough unique data points to generate a minimum convex polygon (MCP). Brook Kleine Dommel is indicated by the bold line.

**Fig. 3.**
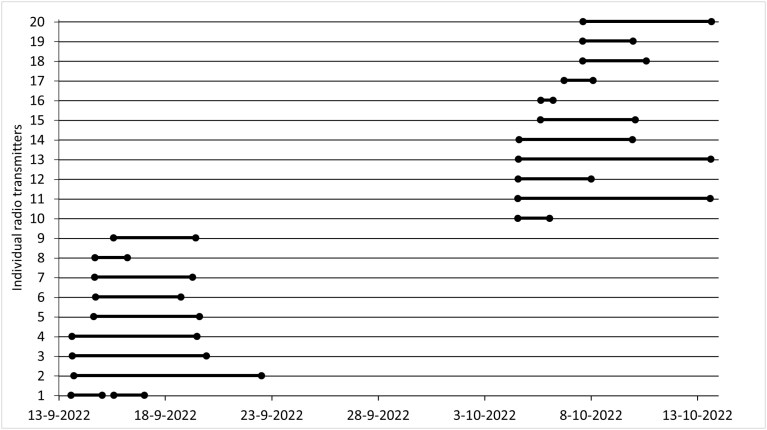
The attachment duration per individual tracked Eurasian Water Shrew (*Neomys fodiens*). The mean transmitter attachment duration was 4.2 days and ranged between 0.6 and 9 days.

The radio tracking survey conducted between September to October 2022 resulted in 1,065 unique GPS locations of Eurasian water shrews, ranging from 5 to 154 locations per individual. After filtering (see Materials and methods section), 332 locations were used in subsequent analyses. The mean number of GPS positions after filtering was 14 to 15 per individual, but varied between 2 and 45 GPS positions. The mean surface of the area used by radio-tracked Eurasian water shrews was estimated through transmitter attachment duration as 1,905 m^2^ but varied among individuals between 90 and 7,844 m^2^, (Fig. 2). The attachment duration of the radio transmitter among 20 individual Eurasian water shrews had a mean of 4.2 (±SD = 2.6) days but ranged from 0.6 to 9.0 days (Fig. 3). Duration of the transmitter attachment per individual correlated with the area it used (Pearson’s *r* = 0.696, df = 17, *P* < 0.001). Among individuals, the used area often partially overlapped with 1 to 4 other Eurasian water shrews and was in 1 case fully situated within the used area of another individual. The centers of occupied areas for individuals were all situated within 100 m of the lowland brook. We observed foraging Eurasian water shrews on brook banks in individuals that were trapped at these locations. However, individuals that were not directly trapped adjacent to the brook were rarely found using a brook bank within their occupied area. No individuals were observed in water (e.g., swimming) or found on the opposite side of the stream, nor was there any indication that Eurasian water shrews were dependent on water for foraging purposes. We observed Eurasian water shrews occupying all available habitats including brook banks, densely vegetated meadows, and riparian forests. Sedges (*Carex* spp.) and reeds often dominated the forest edges (see Supplementary Data SD1 for habitat impressions). Eurasian water shrews often revisited previously used roosts several times per day. They exclusively used underground roosts such as burrow systems of voles (Arvicolinae). Predominantly, roosts had a sheltered position in denser and taller vegetation types such as in complex riparian vegetations, Willow (*Salix* spp.) shrubs, or Alder (*Alnus* spp.) forests. Here, roost entrances were mostly situated under tree trunks or in sedge tussocks. A few times, roost entrances (i.e., vole burrows) were located in densely vegetated meadows.

### Variable selection and model performance.

Spearman rank correlation tests showed that several variables in the initial SDM were highly correlated (*r* > 0.7 or *r* < −0.7; Supplementary Data SD2). First, the variable >10 m density was omitted in the backward selection procedure because it correlated strongly with vegetation height, structural complexity, and canopy cover and was of low importance to the SDM. Subsequently, canopy cover was removed because of its low importance and correlation with the more important variables vegetation height, structural complexity, and 5 to 10 m density. Finally, structural complexity was removed because of its strong correlation with vegetation height. A final model consisted of the following 8 variables: <1 m density; 1 to 5 m density; 5 to 10 m density; vegetation height; variation in vegetation height; forest edge length; open areas; and distance to water. The area under curve (AUC = 0.93), correlation coefficient (COR = 0.76), true skill statistics (TSS = 0.73), and receiver operating characteristic curve (ROC > 0.75) all had values >0.7, while deviance was low (0.82), indicating good fit of the SDM (Supplementary Data SD3).

### Relative variable importance and habitat use.

**Fig. 4.**
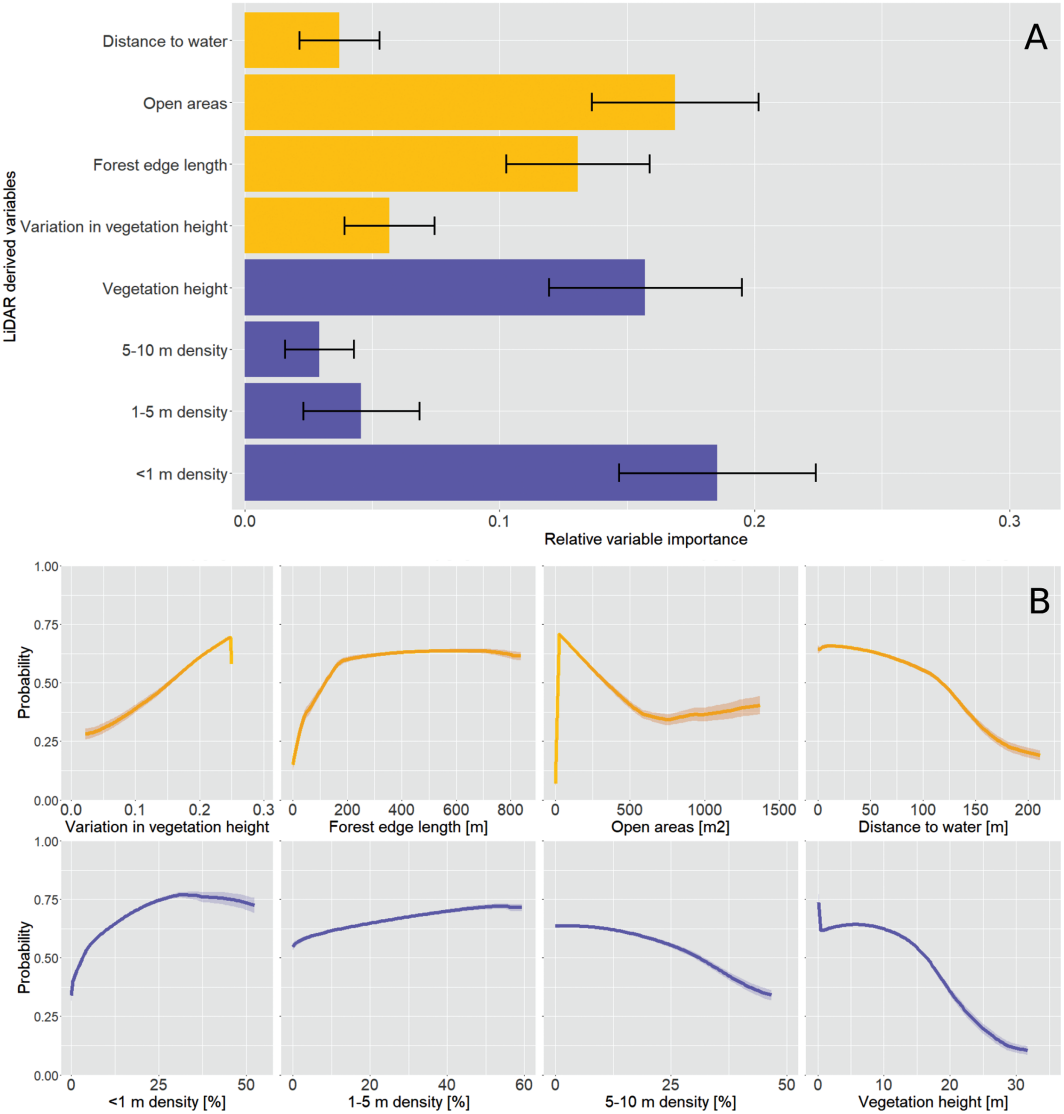
Importance of LiDAR-derived variables for predicting habitat use of Eurasian Water Shrew (*Neomys fodiens*). (A) Relative variable importance, which shows how much each variable contributes to explaining Eurasian Water Shrew habitat use; error bars represent the standard deviation. (B) Response curves displaying the relationship between each variable and predicted habitat use; error bars represent 95% confidence intervals. All figures were calculated using 100 Maxent model runs. The upper four are horizontal and the lower four are vertical variables.

Habitat use of Eurasian Water Shrew was most strongly and positively related to local-level vegetation density <1 m height (0.19; Fig. 4A). At this height layer, the optimal response was at a vegetation density of 20% to 40% with a slight parabolic shape (Fig. 4B). The variable with the second-highest variable importance was the landscape-variable open areas (0.17), which was negatively related to Eurasian Water Shrew habitat use. The response curves show that an increase in open areas leads to lower habitat use for Eurasian water shrews, with the lowest use at around 750 m^2^ of open areas and the highest at around 50 m^2^ (Fig. 4B). The local-level variable vegetation height also had a relatively high importance of around 0.16 with a negative relation to habitat use (Fig. 4A), with highest predicted probabilities between vegetation heights of 0 to 15 m after which habitat use strongly decreased (Fig. 4B). Finally, forest edge length had a positive relation on habitat use and a relative variable importance of 0.15 (Fig. 4A), where an increase in forest edge length up to 200 m increased habitat use, after which the response curves flattened (Fig. 4B). The remaining variables: 1 to 5 m density (positive); 5 to 10 m density (negative); variation in vegetation height (positive); and distance to water (negative) each had a much lower importance as compared to the other variables (i.e., <1 m density, open areas, vegetation height, and forest edge length; Fig. 4A).

## Discussion

### Habitat use.

Knowledge of the fine-scale habitat use by Eurasian Water Shrew is limited as a consequence of its elusive behavior. By combining radio tracking and LiDAR techniques, individual shrew positions were linked to 8 vegetation and landscape variables and analyzed in an SDM. The importance of a relatively high vegetation density below 1 m high for the overall habitat use is in line with our first hypothesis (H1) and previous studies demonstrating that a high cover of herbaceous vegetation is associated with Eurasian Water Shrew occurrence (Carter and Churchfield 2006; Keckel et al. 2014). However, the negative relationship with the variable open areas suggests that habitats with a vegetation below 0.5 m decrease the habitat suitability for Eurasian water shrews. This finding is in accordance with previous studies that demonstrated that low vegetation should not be too short (e.g., short grass; exact height not mentioned) because this will have a negative effect on occurrence of the species (Rychlik 2000; French et al. 2001). Alternatively, Eurasian water shrews prefer herbaceous and sedge-like vegetation, e.g., rushes (*Juncus* spp.), Yellow Loosestrife (*Lysimachia vulgaris*), and Water Mint (*Mentha aquatica*; Supplementary Data SD1). Each grassland is annually mowed for 80%, while 20% is retained (Supplementary Data SD1). We suspect that a lack of cover increases predation risk by, e.g., barn owls (*Tyto alba*), or red foxes (*Vulpes vulpes*; Wołk 1976; Korpimäki and Norrdahl 1989; Love et al. 2000). In addition, food availability on/under the ground (invertebrates) can be low in very open habitats (Duelli et al. 2002; Greenwood et al. 2002; Socher et al. 2012).

The optimal local vegetation height range seems to be between 1 and 15 m. This range covers most of the vegetation present in the area, which is in line with Keckel et al. (2014), who found that Eurasian water shrews accept a wide range of varying parameters when selecting habitats. This pattern suggests that Eurasian water shrews use a wide variety of habitats and will tolerate suboptimal conditions on some habitat characteristics in order to find food or avoid intraspecific competition (Krushinska and Rychlik 1993; Rychlik and Zwolak 2005), and also demonstrates that vegetation up to about 15 m has a positive effect on Eurasian Water Shrew occurrence while taller vegetation, mostly trees, has a negative effect. We found that an increased variation in vegetation height leads to an increased probability of occurrence. Keckel et al. (2014) demonstrated that different age classes of Eurasian water shrews have a different habitat use due to adults that displace subadults and juveniles from optimal habitats (Voesenek and Van Bemmel 1984). It is therefore possible that a higher degree of habitat variation accommodates more different age and sex classes to avoid conflict (Krushinska et al. 1994), associated with a higher probability of occurrence.

The results of this study also highlight a positive effect of forest edge length on Eurasian Water Shrew habitat use, in agreement with our second hypothesis (H2). We found that habitat use increased between a forest edge length of 0 to 200 m, but leveled off between 200 and 800 m. These findings were supported by our field observations, as Eurasian water shrews often showed foraging behavior in forest edges. In our study area, forest edges consisted of several meter-wide zones that were diverse in height and rich in species, which thereby contributed to landscape heterogeneity. The presence of such natural forest edges likely increases the overall habitat use of Eurasian water shrews at a landscape level by providing important shelter and foraging opportunities (Rychlik 2000). A study from Poland also showed the importance of forest edges as microhabitat for Eurasian water shrews which were captured twice as often in ecotones (such as the forest edge) than in Alder forest, but still less often than in adjacent sedge-dominated fields (Rychlik 2000).

In line with our third hypothesis (H3), the SDM identified a decreasing probability of occurrence with increasing distance to water. Other studies also reported that Eurasian water shrews are predominantly bound to riparian habitats adjacent to water (Rychlik 2000; French et al. 2001; Greenwood et al. 2002; Keckel et al. 2014). Nevertheless, the relative importance of the variable distance to water as determined by the AUC was relatively low, meaning that this variable is not a good predictor for the local occurrence of the Eurasian Water Shrew. Indeed, the forest edge habitats in which the shrews were often found were situated relatively far from water on this scale. It is important to note that the scale of the study area might play a role in this result. The study was carried out during a limited time period in a stream valley where small-scale vegetation variables were found to be more important predictors for Eurasian Water Shrew occurrence. The Eurasian Water Shrew is generally described as having a semiaquatic lifestyle (Mendes-Soares and Rychlik 2008; Keckel et al. 2014), meaning that this species is adapted to swim, dive, and forage underwater. Yet, none of the 20 tracked individuals were observed in the lowland brook during the study period. The relatively high importance of forest edge length and low importance of distance to water may be a temporal effect arising from the study period (September to October) and associated prey availability. Even though the Eurasian Water Shrew often feeds on aquatic prey (Haberl 2002), some studies demonstrated that their diet can predominantly consist of terrestrial invertebrates (Churchfield 1985; Kuvikova 1985) and may vary between season, habitat, and prey availability (Churchfield and Rychlik 2006). In a study in a Slovakian Alder forest, to some extent similar to our study area, aquatic prey comprised only 10% of their diet during autumn (Kuvikova 1985). In addition, Niethammer (1978) found that the proportion of consumed terrestrial prey was higher during relatively dry conditions. Our study took place after an extensive period of drought during which the water level of the adjacent lowland brook was low. Consequently, this may have increased the time that the tracked individuals spent foraging on terrestrial prey in forest edge habitats, further away from the brook. Additionally, we did not distinguish the age class of individuals that were equipped with a transmitter—we studied juveniles, subadults, and adults. Our study period coincided with the end of the breeding season, so it could therefore be possible that females were less active. A German study found spatial habitat segregation of Eurasian water shrews between age classes, where adults occupied habitats closer to water but juveniles and subadults were often caught at a further distance to water and in denser vegetation (Keckel et al. 2014). It is possible that we studied relatively many juvenile and subadult individuals that were displaced by adults as a result of intraspecific competition (Voesenek and Van Bemmel 1984), and as a consequence, younger individuals occupied more densely vegetated habitats such as Alder forests and forest edges.

### Robustness of combining telemetry with remote sensing.

Conventional methods to study the presence of Eurasian water shrews are live trapping (Keckel et al. 2014; Kowalski and Rychlik 2018), bait tubing (Greenwood et al. 2002; Resch and Blatt 2013), analyzing owl pellets (Heisler et al. 2015; Gmaj 2021), eDNA, and camera trapping (Verhees et al. 2024). These methods yield presence/absence data of Eurasian water shrews but are limited in terms of studying fine-scale habitat use. For example, baited live trapping, baited tubes, or camera trapping lure animals to specific locations, which may alter natural foraging behavior and habitat use and hence create a location bias (Fidino et al. 2020). In contrast, radio tracking yields high-resolution unbiased location data. Unlike methods relying on predatory animal droppings (e.g., owl pellets) or the placement of baited traps, the use of radio tracking yields actual locations of target species individuals(Dempsey et al. 2015), which makes these data well-suited for studying the habitat use of small, elusive mammals using SDMs. Previous studies have shown the applicability of LiDAR techniques as a robust method to quantify the habitat use and estimate the probability of occurrence of several terrestrial species (Tattoni et al. 2012; Farrell et al. 2013; Acebes et al. 2021; De Vries et al. 2021; Koma et al. 2021). For example, Jaime-González et al. (2017) and Schooler and Zald (2019) found that the inclusion of LiDAR-derived vegetation variables (e.g., structural diversity, and canopy cover) were better predictors of small mammal diversity than field-based variables in a study involving small mammals in a forest habitat. A temporal effect as a result of different recording years between LiDAR and GPS data sets is a common issue in LiDAR studies (Koma et al. 2021). Based on a comparison of satellite imagery (Google Earth) of the past 10 years we argue that the vegetation has not changed substantially during this period which was confirmed by the land manager of the study area, who verified that the majority of the habitats have been preserved in their current state for at least 10 years (Supplementary Data SD1).

The 332 data points used for analyses in this study were collected from 20 animals. While the SDM outputs were reliable, as illustrated by the AUC values of >0.93 (Mandrekar 2010), this may introduce spatial autocorrelation into the experimental design (Dray et al. 2010). We attempted to reduce spatial autocorrelation by including positions of individuals with an interval of >2 h. Possible spatial autocorrelation could have been further reduced by using only 1 position per night, per individual. However, since the mean transmitter attachment duration was 4.2 days (ranging between 0.6 and 9 days), this approach would have made the current analysis impossible due to a low number of data points. An alternative, i.e., increasing the number of experimental animals, raises ethical concerns (e.g., using more animals than necessary, rarity of the species in the Netherlands, risks associated with live trapping). During our study, no mortality occurred in Eurasian water shrews, and 1 animal that lost its transmitter was recaptured and the transmitter was reapplied. This animal had lost 0.5 g in weight (3.3% reduction) after 3 days of being equipped with a transmitter, but similar data on other animals is lacking. Based on our field observations, the shrews showed natural behavior and no visual effects of the transmitter were observed.

Several individuals that were tracked for more than 3 days used distinct habitats and 1 individual used an area of 7.844 m^2^ (Supplementary Data SD1). Lardet (1988) estimated home ranges for Eurasian water shrews near a stream ranging from 77 to 373 m^2^, which were smaller than the areas used by most Eurasian water shrews in our study. In our study, we found a significant positive relationship between the attachment time of transmitter and the home range used by individual Eurasian water shrews. The smaller home ranges as reported by Lardet (1988) are therefore likely an underestimation because the tracking took place within 24 h, while the study area and variables were similar to ours. To acquire more year-round knowledge of Eurasian Water Shrew behavior, follow-up studies could repeat this study in other seasons with different locations and more animals. In addition, more detailed biometric data (e.g., age and sex) about the individual animals should be recorded to determine if, and how, these factors may affect habitat preferences. Future improvements like downsizing of GPS trackers for smaller mammals and increased tracker battery life would further benefit these types of studies (Wild et al. 2022). Such improvements could aid in the simultaneous tracking of animals at different locations and over longer periods. Another advantage of GPS trackers is that the position of an animal can be passively collected which would save on labor costs, reduce disturbance, and minimize spatial autocorrelation. Most importantly, this would result in more accurate GPS positions than can currently be achieved by conventional radio tracking. The results in this study provided a better understanding of the fine-scale habitat use of the Eurasian Water Shrew, which can inform more targeted habitat management, conservation, and restoration of the species. The application of radiotelemetry data combined with LiDAR data, in which many variables can be analyzed together in 1 model, is a promising approach for studying species–habitat relationships of small and elusive terrestrial species such as the Eurasian Water Shrew.

## Supplementary data

Supplementary data are available at *Journal of Mammalogy* online.

**Supplementary Data SD1.** Photographic impressions of the study area.

**Supplementary Data SD2.** Spearman’s rank correlation.

**Supplementary Data SD3.** ROC curve.

gyae146_suppl_Supplementary_Datas_D1_1_D1_5

gyae146_suppl_Supplementary_Data_D2_1

gyae146_suppl_Supplementary_Data_D3_1

## Data Availability

The telemetry data from the current study are available from the corresponding author upon reasonable request.
