# Supplementary material for: Insights into the fine-scale habitat use of Eurasian Water Shrew (Neomys fodiens) using radio tracking and LiDAR
Source: J Mammal. 2025 Jan 10;106(3):549–60. doi: 10.1093/jmammal/gyae146 (PMC13128199; doi:10.1093/jmammal/gyae146)
Supplement: gyae146_suppl_Supplementary_Datas_D1_1_D1_5 [file gyae146_suppl_supplementary_datas_d1_1_d1_5.docx]

**Supplementary Data SD1. Photographic impressions of the study area.**


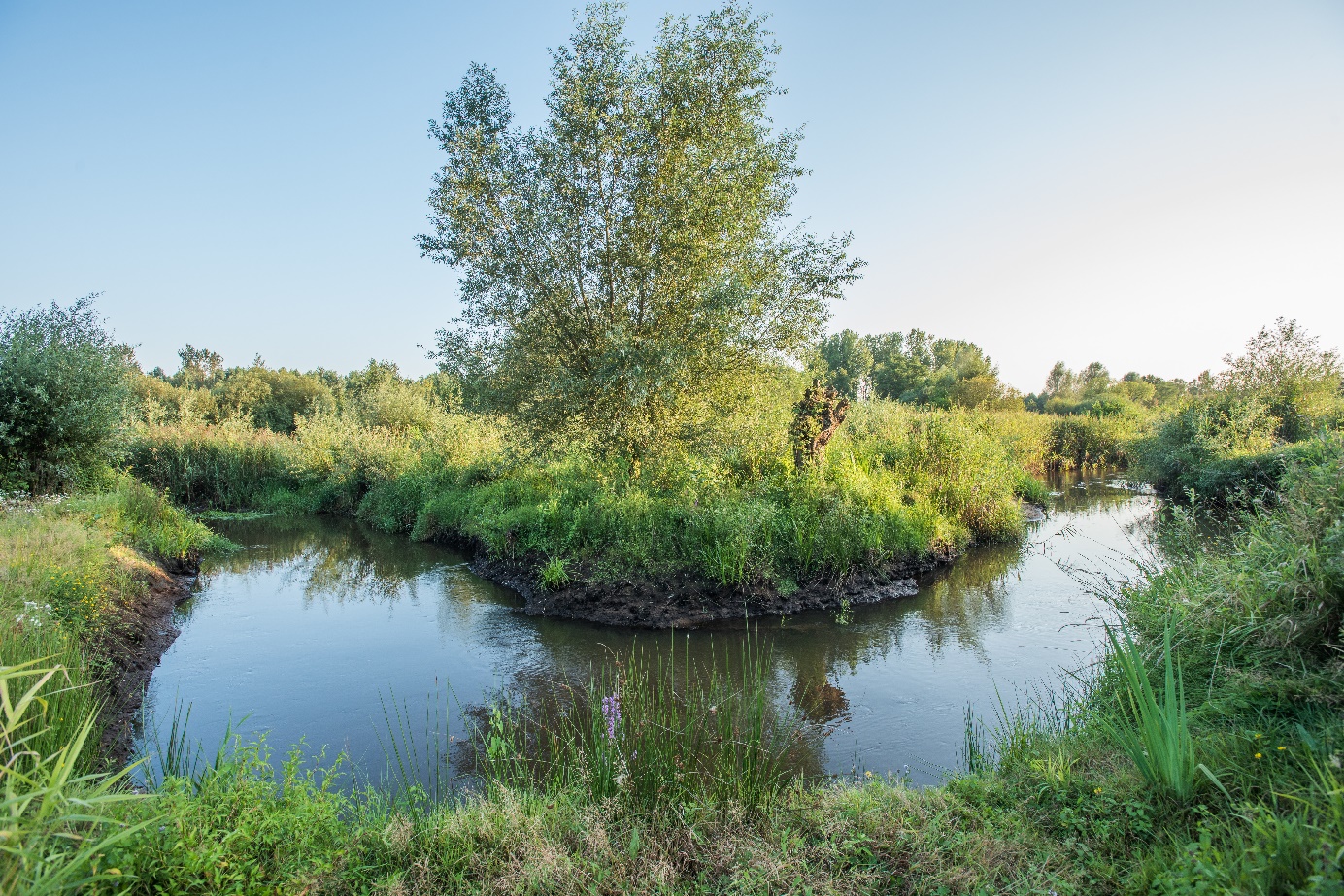


Fig. SD1.1. A meander of the lowland brook (Kleine Dommel) in the central part of the study area. The banks were natural, with a gentle angle and partly covered with several species of herbaceous vegetation and Common Reed (*Phragmites australis*). The regular water level is about 40-80 cm. At the time of the study, there was a drought and the water level was estimated at 10-40 cm. During the study, Eurasian water shrews (*Neomys fodiens*) were observed near the banks of the brook but never in the water. The riparian and submerged vegetations are mowed annually.


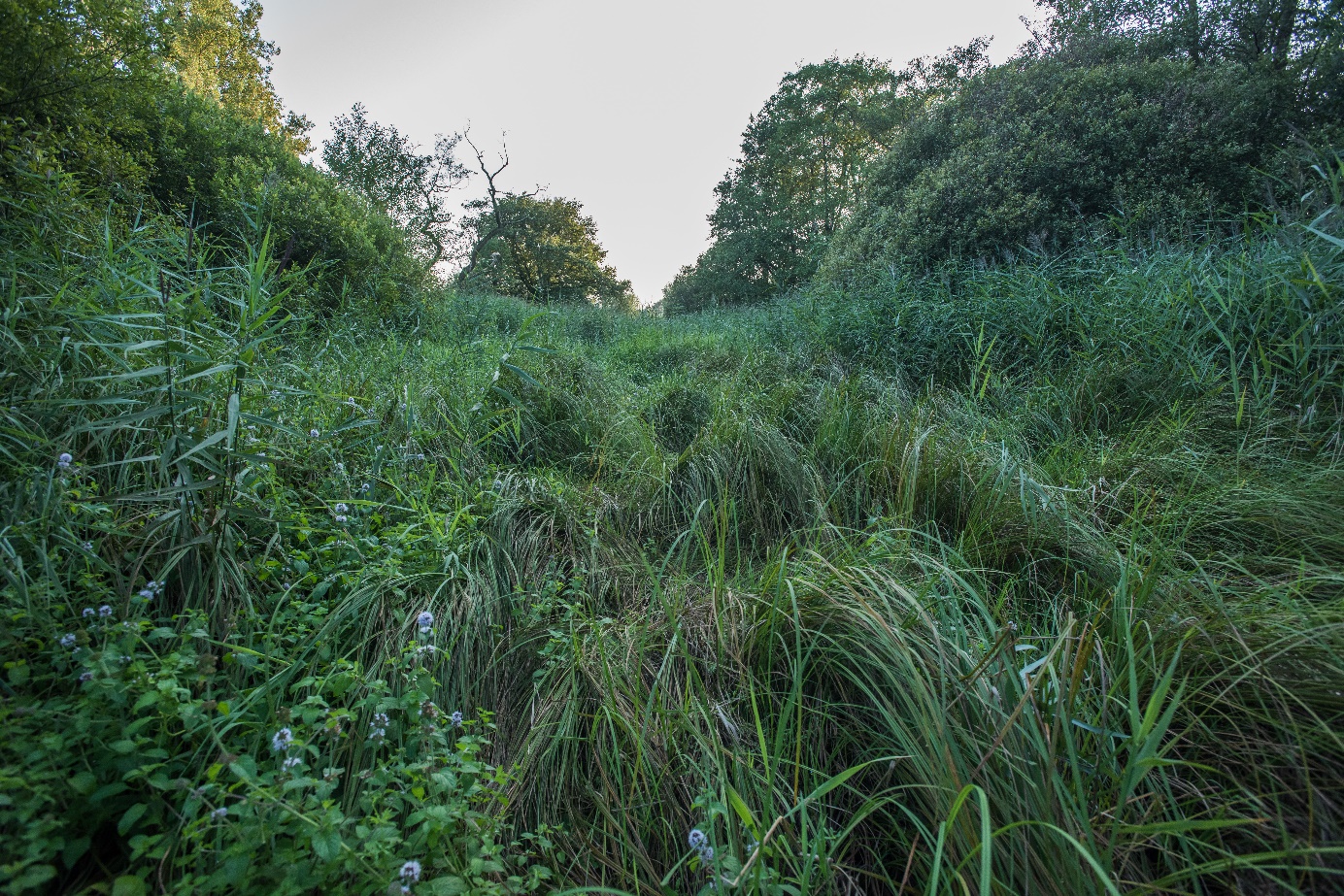


Fig. SD1.2. A field with vegetation <1 m where sedges (*Carex* spp.) dominate and where herbaceous vegetation such as Water Mint (*Mentha aquatica*) also occurs. The field is surrounded by higher vegetations such as Common Reed (*Phragmites australis*), Birch (*Betula* spp.), Alder (*Alnus glutinosa*), and Willow (*Salix* spp.). This site and those with a similar vegetation were frequently used by the Eurasian water shrews (*Neomys fodiens*). Management of such habitats usually takes place once a year by mowing and removal in autumn (mostly September). Each time, 5-10% of the plot area remains unmowed. For such habitats, there are years when it is too wet to mow, preventing management, as was the case here.


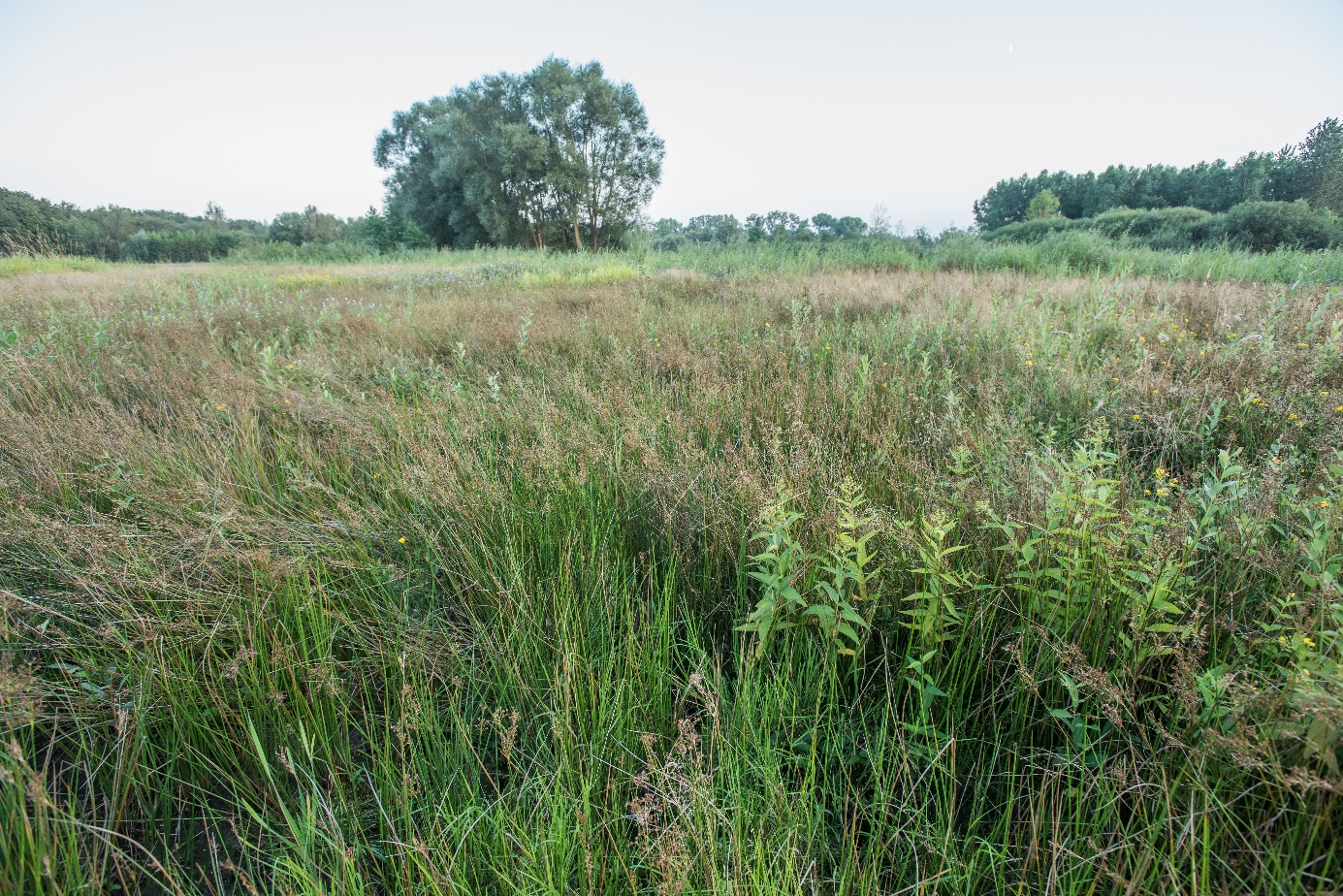


Fig. SD1.3. A field with vegetation <1 m where rushes (*Juncus* spp.) dominate and where herbaceous vegetation such as Yellow Loosestrife (*Lysimachia vulgaris*) and Water Mint (*Mentha aquatica*) also occurs. This site and those with a similar vegetation were frequently used by the Eurasian water shrews (*Neomys fodiens*). Management of such habitats takes place once a year by mowing and removal in autumn (mostly September). Each time, 5-10% of the plot area remains unmowed.


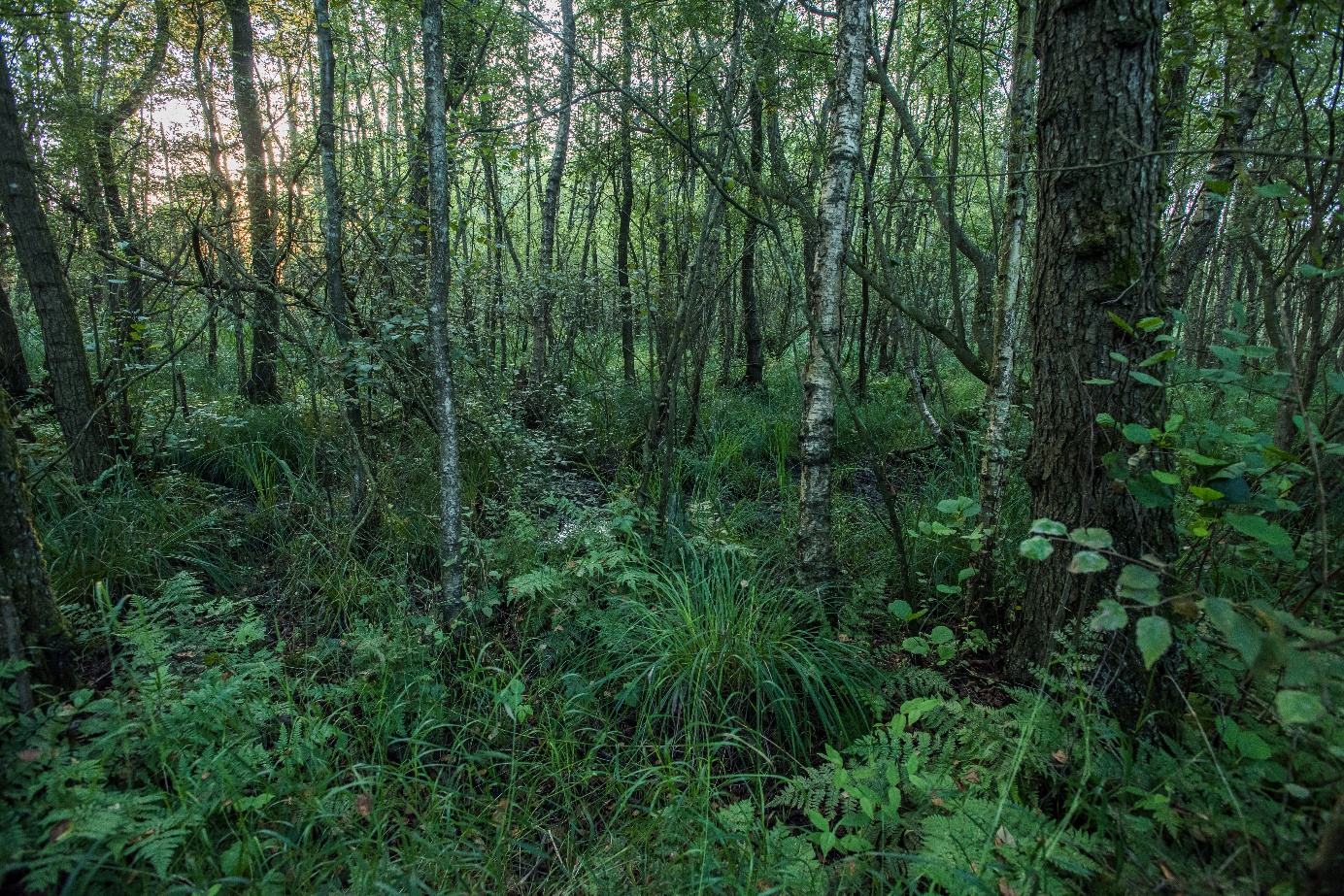


Fig. SD1.4. An example of a complex riparian vegetation with mainly sedges (*Carex* spp.) in the <1 m understory and, to a lesser degree, wood ferns (*Dryopteris* spp.). Higher vegetations include Birch (*Betula* spp.), Alder (*Alnus glutinosa*), and Willow (*Salix* spp.). This site and those with a similar vegetation were frequently used by the Eurasian water shrews (*Neomys fodiens*). Also, underground roosts were predominantly found in these types of vegetation. This type of habitat is not managed.


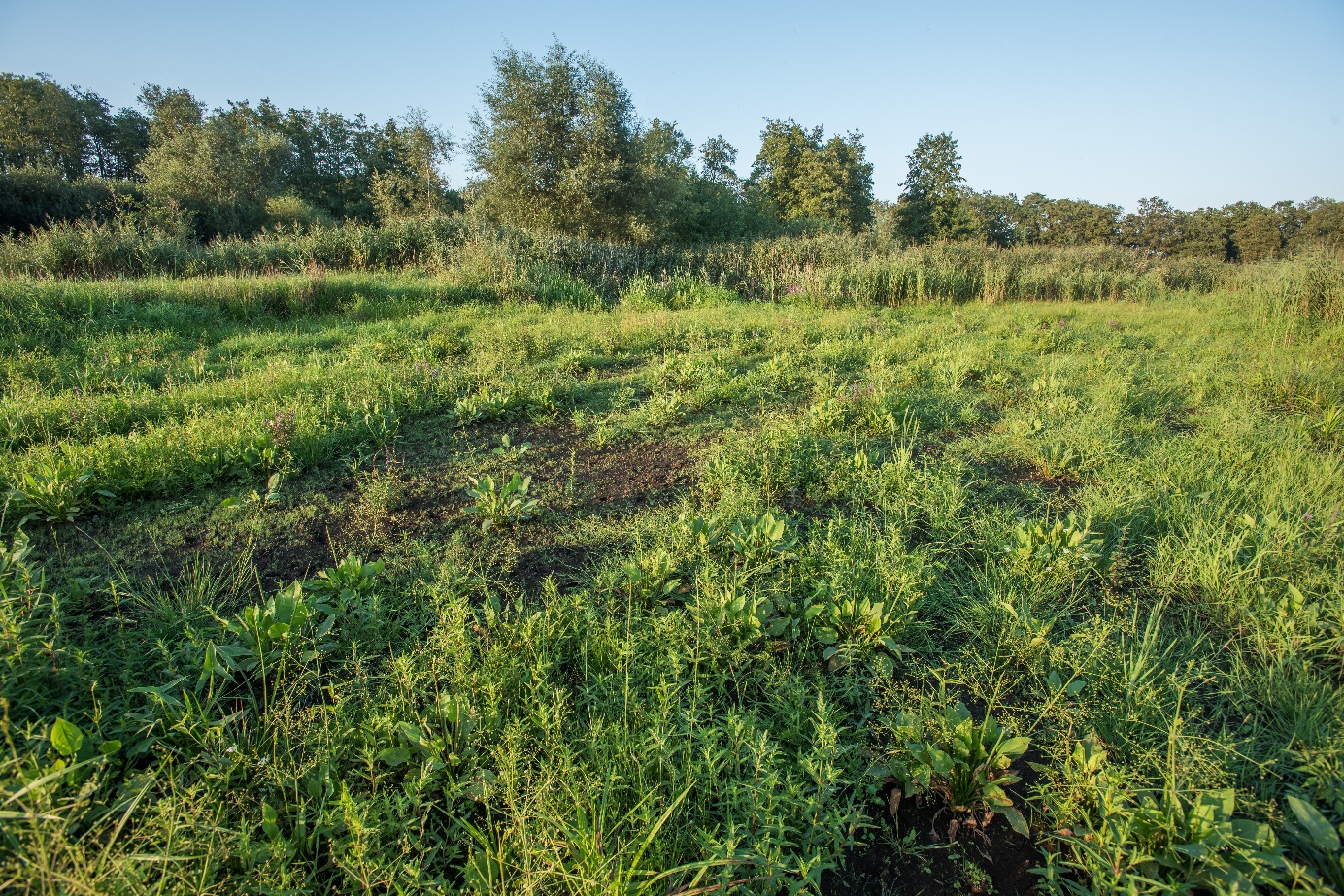


Fig. SD1.5. A field with less dense vegetation <1 m consisting of Common Water-Plantain (*Alisma plantago-aquatica*), Purple Loosestrife (*Lythrum salicaria*) and several grasses (Poaceae indet.). This site and those with a similar vegetation were rarely used by the Eurasian water shrews (*Neomys fodiens*). Management of such habitats takes place once a year by mowing and removal in autumn (mostly September). Each time, 5-10% of the plot area remains unmowed.
