# Supplementary material for: Insights into the fine-scale habitat use of Eurasian Water Shrew (Neomys fodiens) using radio tracking and LiDAR
Source: J Mammal. 2025 Jan 10;106(3):549–60. doi: 10.1093/jmammal/gyae146 (PMC13128199; doi:10.1093/jmammal/gyae146)
Supplement: gyae146_suppl_Supplementary_Data_D2_1 [file gyae146_suppl_supplementary_data_d2_1.docx]

**Supplementary Data SD2. Spearman’s rank correlation.**


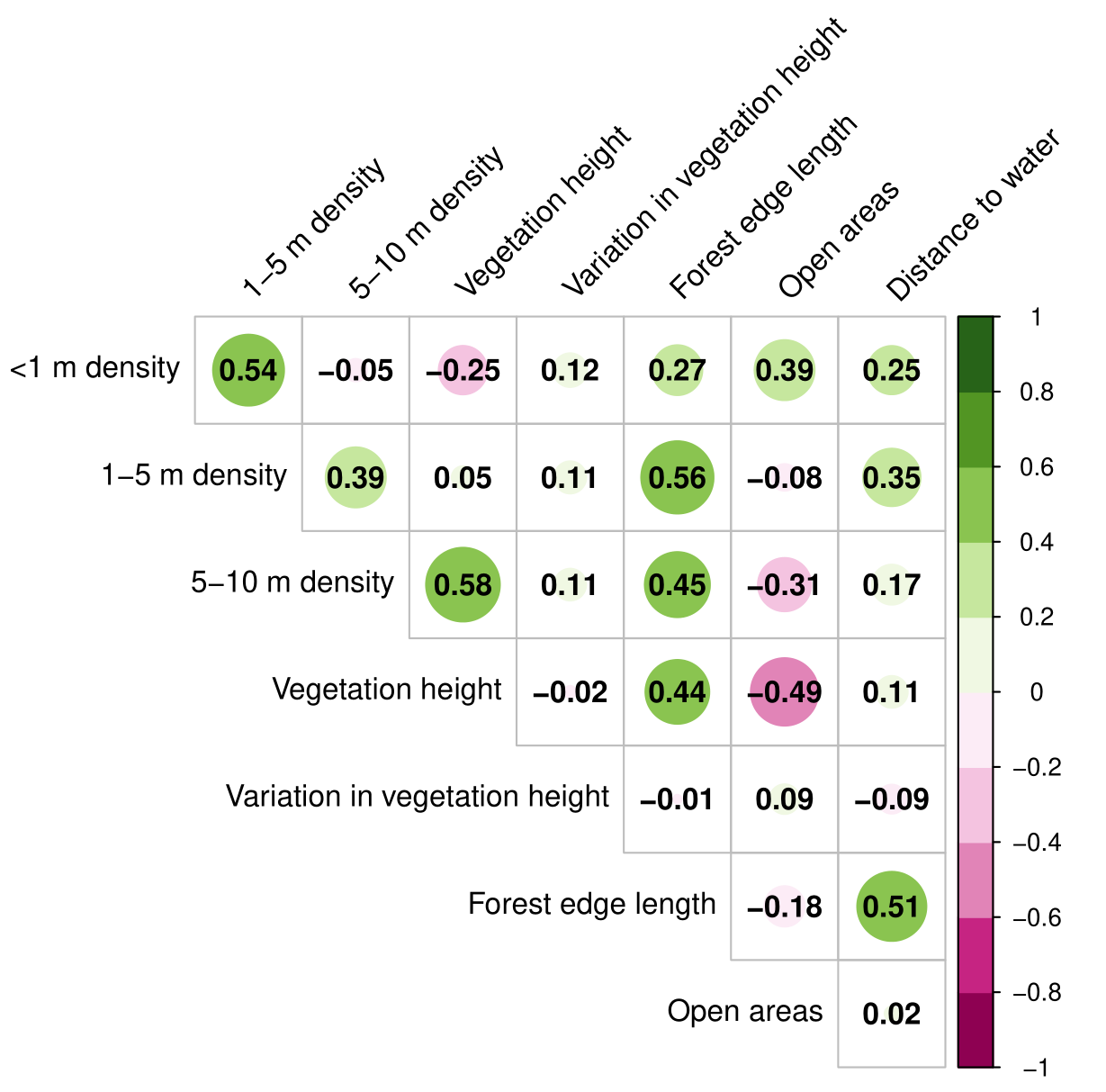


Fig. SD2.1. Spearman’s rank correlation measure for all calculated variables. Spearman’s values of *r* > 0.7 or *r* < -0.7 demonstrate multicollinearity between variables. Light colors show a low correlation and darker colors show a higher correlation between variables.
