# Supplementary material for: Insights into the fine-scale habitat use of Eurasian Water Shrew (Neomys fodiens) using radio tracking and LiDAR
Source: J Mammal. 2025 Jan 10;106(3):549–60. doi: 10.1093/jmammal/gyae146 (PMC13128199; doi:10.1093/jmammal/gyae146)
Supplement: gyae146_suppl_Supplementary_Data_D3_1 [file gyae146_suppl_supplementary_data_d3_1.docx]

**Supplementary Data SD3. ROC curve.**


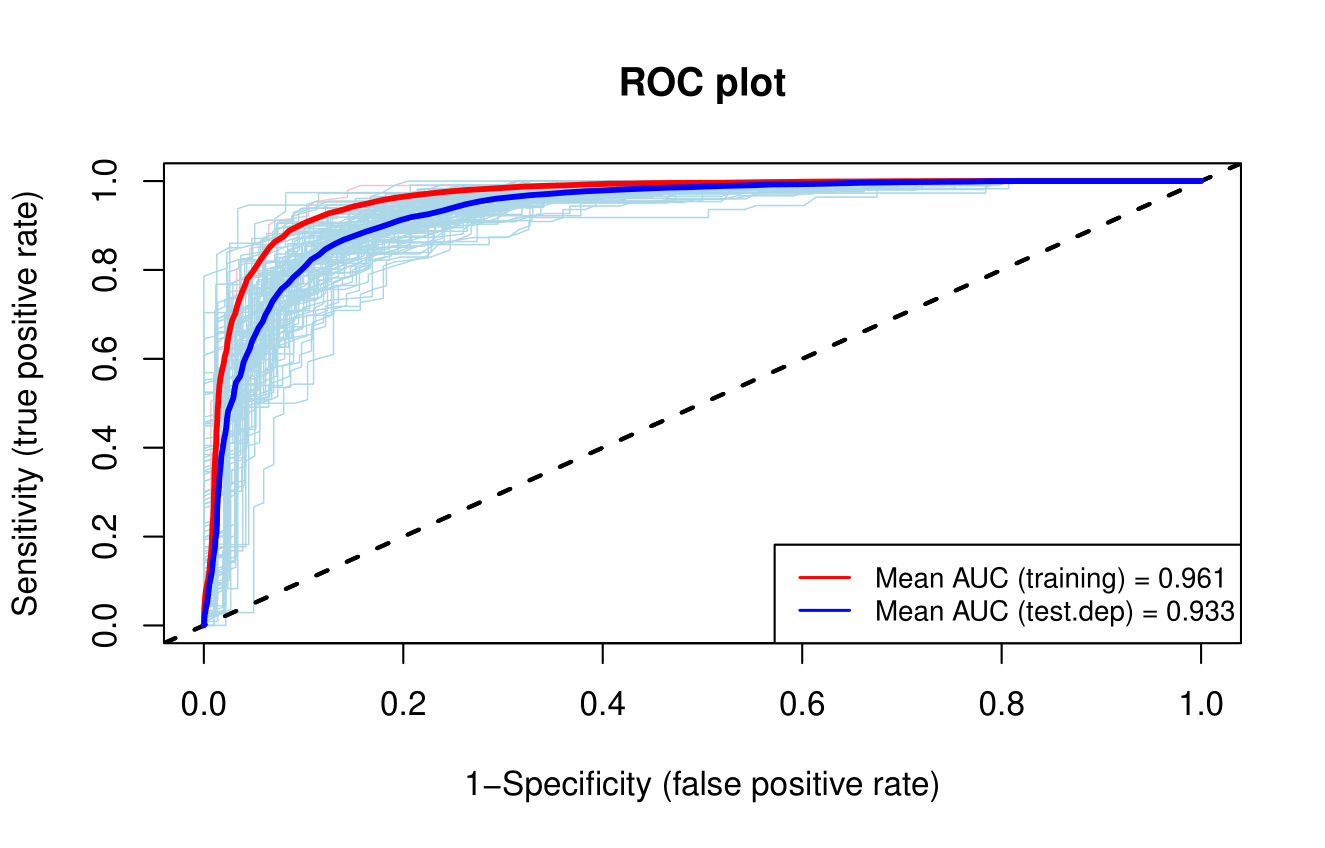


Fig. SD3.1. Receiver Operating Characteristics (ROC) curve for the SDM analyzed with a Maxent analysis and root n=100. Species distribution model includes the variables: <1 m density, 1-5 m density, 5-10 m density, vegetation height, variation in vegetation height, forest edge length, open areas, distance to water. Thin blue lines indicate 100 validation runs. The bold blue line shows the mean of these 100 runs.
